# Online hyphenation of size‐exclusion chromatography and gas‐phase electrophoresis facilitates the characterization of protein aggregates

**DOI:** 10.1002/elps.202100018

**Published:** 2021-03-10

**Authors:** Victor U. Weiss, Natalia Denderz, Günter Allmaier, Martina Marchetti‐Deschmann

**Affiliations:** ^1^ Institute for Chemical Technologies and Analytics TU Wien (Vienna University of Technology) Vienna Austria

**Keywords:** MacroIMS, nES DMA, nES GEMMA, Protein aggregates, Size‐exclusion chromatography

## Abstract

Gas‐phase electrophoresis yields size distributions of polydisperse, aerosolized analytes based on electrophoretic principles. Nanometer‐sized, surface‐dry, single‐charged particles are separated in a high laminar sheath flow of particle‐free air and an orthogonal tunable electric field. Additionally, nano Electrospray Gas‐Phase Electrophoretic Mobility Molecular Analyzer (nES GEMMA) data are particle‐number based. Therefore, small particles can be detected next to larger ones without a bias, for example, native proteins next to their aggregates. Analyte transition from the liquid to the gas phase is a method inherent prerequisite. In this context, nonvolatile sample buffers influence results. In the worst case, the (bio‐)nanoparticle signal is lost due to an increased baseline and unspecific clustering of nonvolatile components. We present a novel online hyphenation of liquid chromatography and gas‐phase electrophoresis, coupling a size‐exclusion chromatography (SEC) column to an advanced nES GEMMA. Via this novel approach, it is possible to (i) separate analyte multimers already present in liquid phase from aggregates formed during the nES process, (ii) differentiate liquid phase and spray‐induced multimers, and (iii) to remove nonvolatile buffer components online before SEC–nES GEMMA analysis. Due to these findings, SEC–nES GEMMA has the high potential to help to understand aggregation processes in biological buffers adding the benefit of actual size determination for noncovalent assemblies formed in solution. As detection and characterization of protein aggregation in large‐scale pharmaceutical production or sizing of noncovalently bound proteins are findings directly related to technologically and biologically relevant situations, we proposed the presented method to be a valuable addition to LC‐MS approaches.

**Abbreviations**
EM: electrophoretic mobility
GEMMA: gas‐phase electrophoretic mobility molecular analysis
Lpm: liters per minute
MW: molecular weight
nDMA: nano differential mobility analyzer
nES: nano electrospray
NH_4_OAc: ammonium acetate
VLP: virus‐like particle

## 1 Introduction

For pharmaceutical applications, the aggregation behavior of proteins and proteinaceous analytes is of importance as corresponding aggregates are often no longer active biologicals in formulations. In addition, and in the worst case, aggregate formation does not only reduce the efficacy of a drug but also leads to side effects [1]. Hence, the characterization of the aggregation behavior of compounds is a necessary but not always easily feasible task.

Gas‐phase electrophoresis on a nano Electrospray Gas‐phase Electrophoretic Mobility Molecular Analyzer (nES GEMMA) [2] separates single‐charged, surface‐dry, native, and intact analytes in the gas phase according to their electrophoretic mobility diameter (EM diameter). In case of spherical particles, the EM diameter corresponds to the analyte diameter. Analytes are electrosprayed from a volatile electrolyte solution, followed by drying of droplets in the gas phase. Concomitantly, charge equilibration occurs in a bipolar atmosphere induced by a ^210^Po α‐particle source, a soft X‐Ray charger, a corona discharge or similar [3, 4]. Obtained particles are surface dry and mostly neutral or single charged. Usually, for particles in the size range below 50 nm EM diameter, the fraction of multiple‐charged particles is low and can be disregarded [5, 6]. Particles are subsequently introduced to the differential mobility analyzer (DMA) part of the instrument. There, a combination of a constant high laminar sheath flow of particle‐free air and an orthogonal, tunable electric field enables separation of single‐charged analytes only according to their EM diameter. Size‐separated particles are then counted after having induced nucleation in a supersaturated atmosphere of either *n*‐butanol or water. Via this setup, particle number concentrations are detectable in accordance with recommendations of the European Commission regarding nanoparticle characterization (2011/696/EU from October 18th, 2011). Depending on the geometry of the applied DMA as well as on settings of the high laminar sheath flow, (bio‐)nanoparticles – for instance, liposomes [7, 8, 9, 10], viruses, and virus‐like particles (VLPs) [11, 12, 13, 14, 15], proteins [16, 17, 18], organic, or inorganic nanoparticles [19, 20] – from very few up to several hundred nanometers EM diameter can be analyzed. For certain DMAs, even larger analytes can be targeted. Resulting spectra relate EM diameter values to particle counts or particle concentration values.

In literature also other names for the same instrumental setup can be found and have to be regarded, that is, ES DMA [21], scanning mobility particle sizer [22] or LiquiScan ES (company‐provided name). In this work, a next‐generation nES GEMMA instrument, a so‐called MacroIMS (for some time the name for such a commercially available setup provided by TSI Inc.) was used. In this advanced setup, a soft X‐Ray charge conditioning is used instead of a ^210^Po α‐particle source, the geometry of the nES unit is different [23] and a water‐based nucleation and particle detection system is installed.

The power of gas‐phase electrophoresis to characterize aggregational behavior of proteins in solution has been shown from early on. Already in 2001, Bacher et al. described the applicability of nES GEMMA in protein research [24] showing that the EM diameter correlates with the molecular weight (MW) of standards, for example, for proteins. Only recently this has been advanced for viruses [14], VLPs [15], polysaccharides [25], or other substance classes showing its potential to access a MW range not easily covered by conventional mass analyzers. Measurements of noncovalent interactions resulting in large protein assemblies were described by Blake and Blake or Laschober et al. for antibody/antigen binding [26, 27]. Bereszczak et al. presented VLP/antibody fragment interactions in relation to native MS [11], Havlik et al. published the analysis of unspecific aggregation of VLPs [13] and Engel et al. [28] described the analysis of specific lectin/glycoprotein complexes.

Likewise, nES‐induced multimer formation for higher analyte concentrations occurs. In case the number of analyte molecules exceeds the number of formed electrolyte droplets at the tip of the nES capillary, more than one analyte molecule can be found per droplet. Subsequently, after solvent evaporation, these particles are detected as corresponding multimers interfering with the detection of liquid‐phase multimerization and leading, in this respect, to artefacts from the measurement process.

Multimers are easily formed in the nES droplets and they tend to be a source of misinterpretation [17] and debate ‐ are analytes aggregating in the liquid or the gas phase? It has been an ongoing discussion, especially in the community studying noncovalent protein assemblies, to undoubtedly show that measured protein aggregates are actually already formed in liquid and are therefore biologically relevant or to know that they are formed during the nES process. We now present an online hyphenation of SEC with gas‐phase electrophoresis, a combination of two complementary dimensions of separation, to overcome this problem. The SEC–advanced nES GEMMA hyphenation allows to separate sample components in solution according to their hydrodynamic particle diameter prior an electrophoretic separation according to the surface‐dry particle diameter in the gas phase. Hence, aggregates already formed in the liquid phase can clearly be differentiated from nES induced ones.

In addition to the differentiation of gas‐ and liquid‐phase particle multimerization, our novel method hyphenation can also be employed to carry out online sample desalting. By implementing a short electrophoretic separation in the nES capillary, we have already demonstrated the importance of low salt concentrations [29]. In continuation of this work, we also discuss the potential of the presented SEC–next‐generation nES GEMMA hyphenation for online sample desalting.

## 2 Materials and methods

### 2.1 Chemicals and reagents

Ammonium acetate (≥99.99%) and ammonium hydroxide (ACS reagent) as well as thyroglobulin (bovine), albumin (from chicken egg white aka ovalbumin), μ‐globulins aka IgG (bovine), and hemoglobin (bovine) were obtained from Sigma–Aldrich (Steinheim, Germany). The proteins were applied each at 0.5 mg/mL in ammonium acetate (NH_4_OAc, 40 mM, pH 6.7). Sodium chloride (pro analysis, p.a., up to 15 mM) was purchased from Merck (Darmstadt, Germany) and spiked to samples. Water was of Millipore grade (Burlington, MN, USA ‐ 18.2 MΩ cm resistivity at 25°C).

### 2.2 Instrumentation

For SEC, a Hitachi L‐2000 instrument (VWR, Vienna, Austria) employing a TSKgel G3000SWXL column (7.8 × 300 mm) packed with 5 μm particles was used (Tosoh Bioscience, Griesheim, Germany). NH_4_OAc (40 mM, pH 6.7) was filtered (Corning 0.22 μm polyether sulphone filters) and employed as isocratic mobile phase at 0.7 mL/min. Twenty microliters of each sample was injected into the system. Analytes were detected at 210 nm UV absorption. Subsequent data analysis was carried out employing the HyStar software (version 1.3, Bruker, Billerica, MA, USA). Gas‐phase electrophoresis was carried out on a MacroIMS system (TSI Inc., Shoreview, MN, USA) consisting of a nES unit with a soft X‐Ray charger (model 3482), a classifier (model 3082) equipped with a nano Differential Mobility Analyzer (nDMA, model 3085), and a water‐based, ultrafine condensation particle counter (model 3788). A fused silica capillary, 25 μm inner diameter, with a tip (TSI Inc.) was used for generation of the nES. EM diameter values from 2 to 44 nm were scanned by voltage variation (11 s scan time). Three seconds time for readjustment of the voltage (retrace time) and 1 s purge time were additionally selected leading to detection of particles in 163 channels. Thirty liters per minute (Lpm) was chosen as sheath flow for nDMA measurements. Air flow (1.5 Lpm) and 0.1 Lpm CO_2_ flow were applied to generate a stable nES conus.

### 2.3 Online hyphenation of SEC and gas‐phase electrophoresis

**Figure 1:**
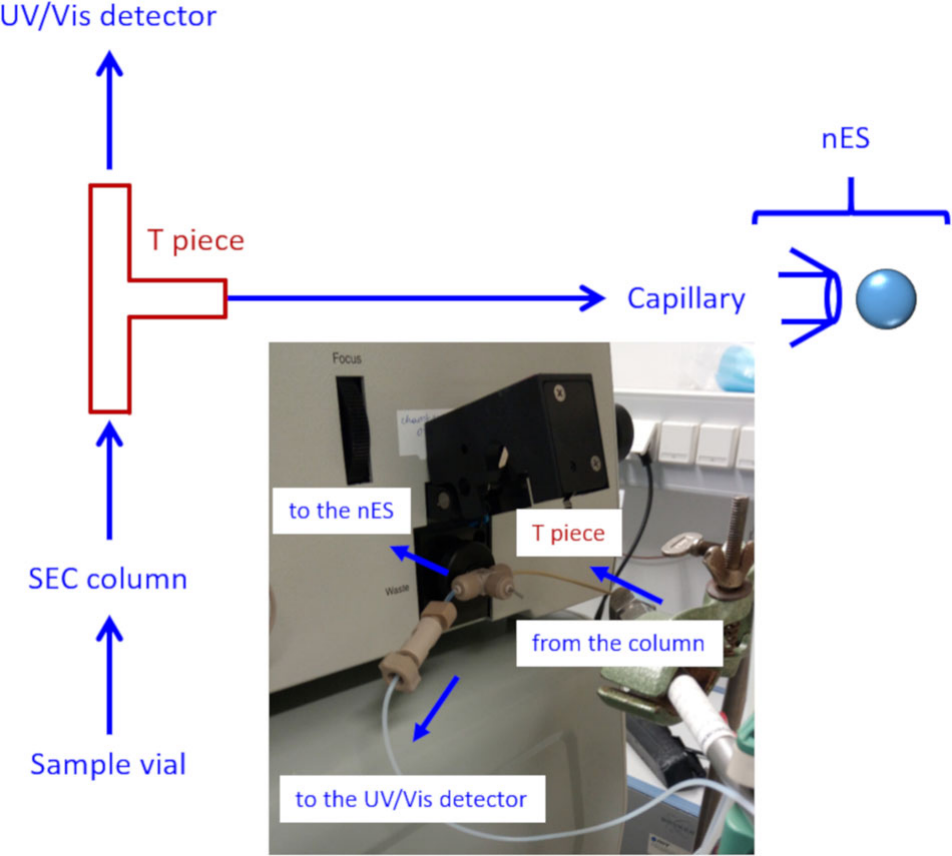
Schematic drawing of the SEC–MacroIMS concept. The inset depicts a photograph of the actual setup.

The flow eluting from the SEC column was split by application of a T‐piece as supplied by TSI as part of the MacroIMS setup. The perpendicular flow led to the nES source of the MacroIMS system, whereas the direct flow led to the UV detector of the chromatographic system (see Fig. 1). An estimated flow of several microliter per minute (approx. 1% of the overall flow) at the tip of the fused silica capillary of the nES source led to a stable sample spray.

### 2.4 Data analysis

Results were plotted in Origin (OriginPro, version 9.1.0, 32 bit from OriginLab Corporation, Northampton, MA, USA). Gauss peaks were fitted to spectra. An Adj. *R*² ≥ 0.75 value was chosen as limit to include numbers to data evaluation. The low Adj. *R*² value was sometimes reached due to low analyte concentrations, especially for aggregates, and hence, low peak heights.

## 3 Results and discussion

**Figure 2:**
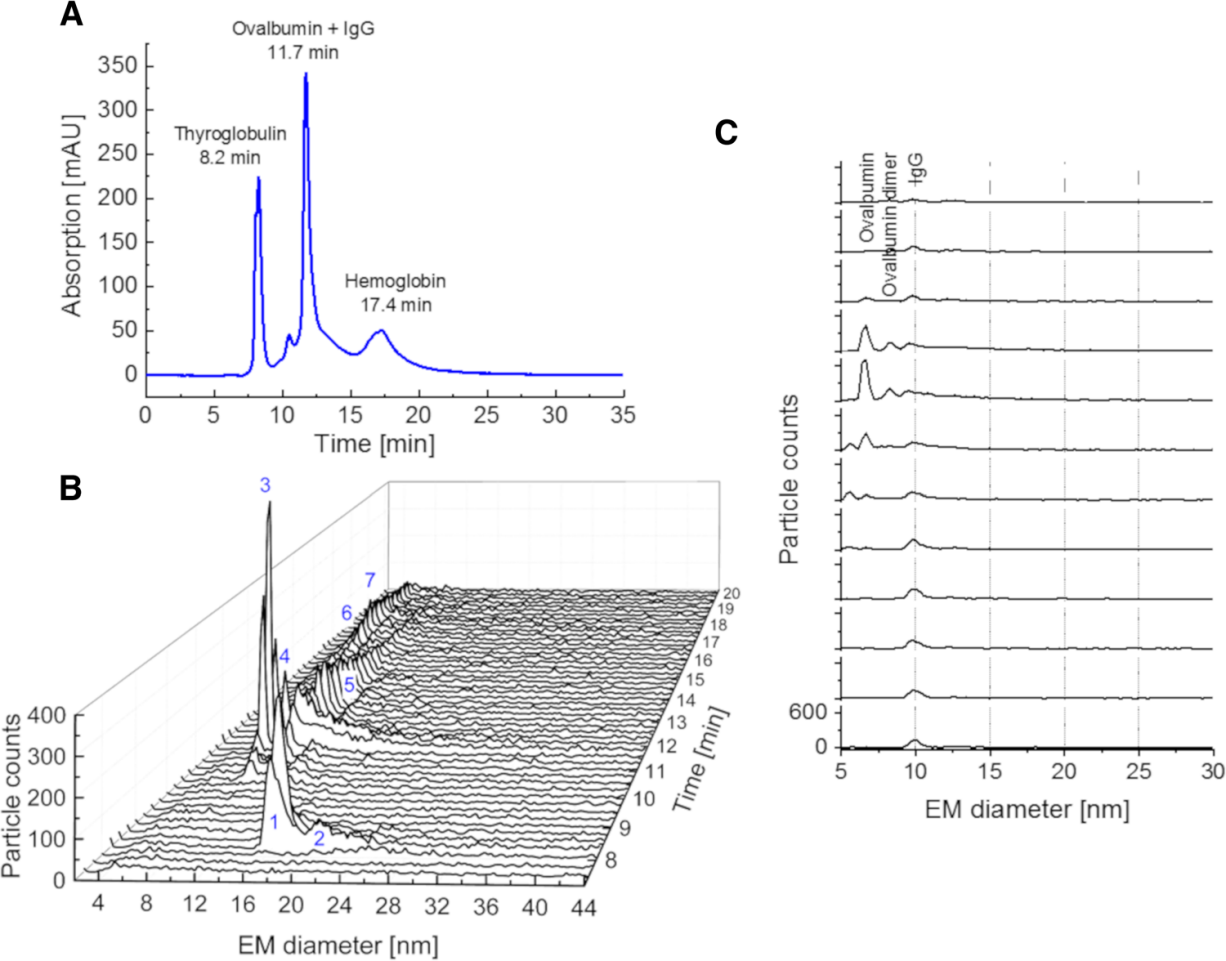
Tyroglobulin, IgG, ovalbumin, and hemoglobin were used for a proof‐of‐concept study using SEC on a TSKgel G3000SWXL column for protein separation and UV detection in parallel to gas‐phase electrophoresis. Proteins could only partially be separated as monitored at 210 nm (A). Especially the separation of ovalbumin and IgG was not feasible. Gas‐phase electrophoresis on a MacroIMS system was employed as second dimension of separation (B). Signals for tyroglobulin monomer (1), tyroglobulin dimer (2), ovalbumin monomer (3), ovalbumin dimer (4), IgG monomer (5), hemoglobin tetramers, (6, biological active form), and octamers (7) were separated. Details of Figure (B) are presented in (C): 11.00–13.75 min SEC elution time is shown. A clear separation of ovalbumin monomers, dimers, and the IgG monomer is found.

In our proof‐of‐concept study, we combined for the first time SEC with an advanced nES GEMMA instrumentation (MacroIMS) in an online hyphenation strategy applying a set of standard proteins of different MW; these proteins are also known for their potential of aggregation. Hemoglobin (approx. 30 kDa – native dimer), ovalbumin (approx. 44 kDa), IgG (approx. 147 kDa), and thyroglobulin (approx. 660 kDa – native dimer) were chosen. The SEC eluate was split directly after the SEC column to monitor protein elution via gas‐phase electrophoresis as a second dimension of separation and in parallel on a conventional UV/Vis detector for reference (see Fig. 1). As shown in Fig. 2, different proteins could (at least partially) be separated prior to gas‐phase electrophoresis based on their hydrodynamic size/shape in the liquid phase. Thyroglobulin and hemoglobin elute at different time points from the SEC column and reach the nES capillary tip (Fig. 2A). By this, the method inherent formation of unspecific gas‐phase aggregates during the nES process can be significantly reduced due to a lower protein amount electrosprayed at the same time (Fig. 2B). However, as observed in the case of ovalbumin and IgG, for some analytes, the SEC separation was not efficient enough under the given conditions (Fig. 2A). Nevertheless, the potential of gas‐phase electrophoresis to be used as a second dimension of separation allowed for an unambiguous detection of ovalbumin (monomer and dimer) besides IgG (monomer) because of their different EM diameters (Figs. 2B and 2C for better visualization).

### 3.1 Are multimers formed in liquid phase identical to gas phase‐based aggregates?

Based on the initial experiments proving the feasibility of online hyphenating SEC and the MacroIMS setup, we subsequently focused on the investigation of analyte multimerization. Given an analyte is electrosprayed at a sufficiently high concentration, its statistical distribution in droplets formed during the nES process will lead to the observation of concentration dependent, unspecific aggregation. Variation of the analyte concentration will result in changes in the observed multimer pattern, which can be seen upon relative plotting of obtained spectra (normalized to the main peak of each spectrum). In MS, such aggregates enable calibration of instruments for a broad mass‐to‐charge range with only a limited number of calibration standards. For nES GEMMA, essentially the same approach was employed by Bacher et al. [24] to establish an EM diameter/MW correlation for proteins. Based on such a correlation, the MW of a protein in question can be calculated based on its EM diameter value. However, data points resulting from spray‐induced (i.e., concentration dependent), unspecific analyte aggregation slightly deviated from the protein monomer‐based correlation. Taking account of this observation, we reasoned that multimer formation in the gas phase can be considerably different from liquid‐phase multimerization. Potentially denser aggregates reduce the EM diameter of particles in the gas phase then when compared to multimerization in buffer solutions.

**Figure 3:**
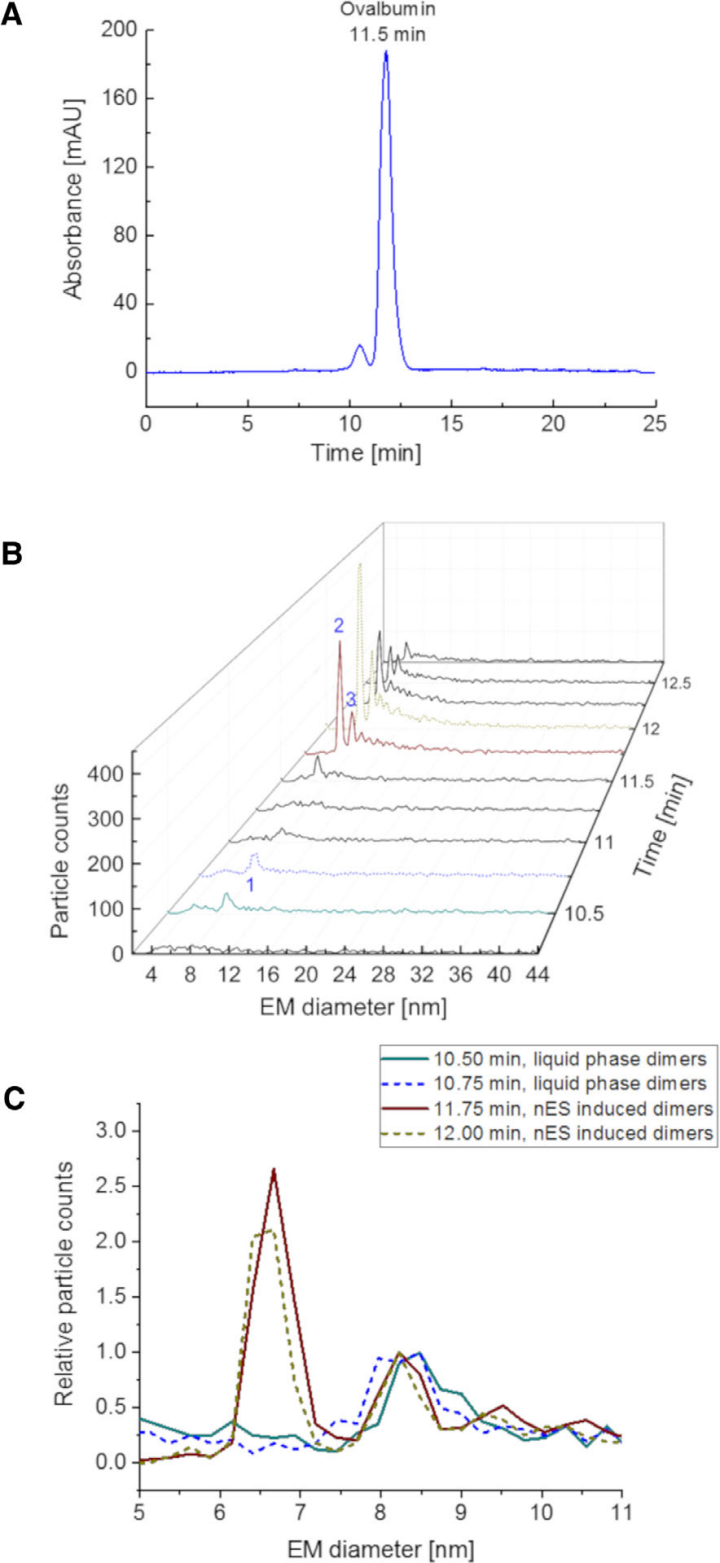
Separation of ovalbumin dimers from monomers via SEC monitored at 210 nm (A) and in parallel by gas‐phase electrophoresis using the MacroIMS system (B) – ovalbumin dimers found in liquid phase (1), monomers (2), and ovalbumin gas‐phase‐induced dimers (3) are labelled. As demonstrated, dimer EM diameter values were different for gas‐phase‐induced dimers from the nES process (11.75 and 12.00 min, red and dark yellow line, respectively) and multimers existing actually in the liquid phase (10.50 and 10.75 min, green and blue line, respectively) (C). Spectra were normalized to dimer peaks.

Indeed, as demonstrated in Fig. 3 employing ovalbumin as protein standard this differentiation can be observed. The separation of dimers already formed in the liquid phase from protein monomers is possible via SEC (Fig. 3A). Subsequent gas‐phase electrophoresis (Fig. 3B) is then able to measure such multimeres, but of course also those formed in the gas phase. Overlaying spectra showing liquid phase formed and nES‐based dimers (Fig. 3C) reveals slight but statistical valid differences between these two species. Liquid‐phase formed dimers were detectable at 8.34 ± 0.06 nm EM diameter, whereas nES‐based dimers exhibited an EM diameter of 8.24 ± 0.02 nm. In both cases, values were taken from at least *n* = 7 spectra from at least *n* = 4 analyses with/without sodium chloride addition, Gaussian peaks were fitted to data points, sodium chloride seemingly had no impact on observed EM diameter values after SEC–MacroIMS analysis, see below. This small but significant difference possibly indicates structural variations between dimers formed in liquid phase and in nES‐based dimers. The dimer formed in the buffer system exhibits a slightly larger diameter, a fact that can be contributed to solvation of the interacting monomers. Such solvent molecules might be part of the final protein dimer arrangement. For the gas‐phase multimers, solvent molecules are seemingly not a part of the three‐dimensional arrangement. This can be explained by the solvent removal from protein surfaces during the drying of droplets, following the nES process. The two remaining, surface‐dry proteins are therefore able to form a denser three‐dimensional structure resulting in an EM diameter difference of –0.1 nm.

Interestingly, liquid‐phase dimers are not detected as completely homogeneous species. This is reflected in EM diameters of fitted Gauss peaks. In consecutive spectra, the obtained EM diameter was always lower (up to 3%) for spectra recorded at later time points. This trend indicates either differences between dimers formed in liquid phase (possibly unspecific aggregation) and/or partial separation of dimers also according to their shape via SEC (Fig. 3C). This trend is also reflected in the higher standard deviation for liquid‐phase dimers when compared to nES‐induced aggregates. For the latter, the same effect is observed to a much lesser extent.

### 3.2 Can peak shapes from gas‐phase electrophoresis be improved?

**Figure 4:**
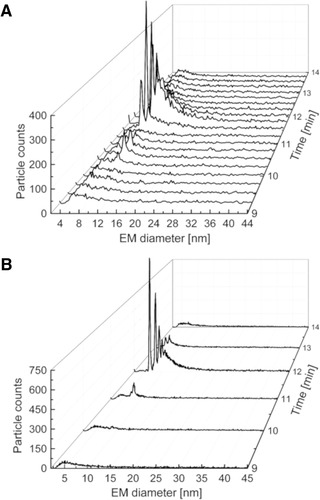
Comparison of SEC/MacroIMS separations obtained for ovalbumin measured at 11 s (A) to 56 s (B) scan time in the nDMA, respectively. While signal shape improved in terms of signal height and width, the longer scanning times led to a loss in data points over time leading to a significant loss of resolution for the SEC separation.

In order to improve peak shapes by reducing peak widths, longer nDMA scan times were tested. The term “scan time” refers to the time in which the electric field of the nDMA is adjusted to allow for separation of surface‐dry particles. At the same time, peak intensities increase as the dwell time per detector channel is positively influenced. Previous studies (unpublished material) directly analyzing ovalbumin without any prior separation have revealed that an increase in scan time led to an approximately twofold increase in signal heights and to a notably reduced peak width (approx. factor of two for the full width at half maximum). Here, we present data on ovalbumin after SEC separation showing the same effect (Fig. 4). Signal intensity and peak width was positively influenced when increasing the scan time from 11 (Fig. 4A) to 56 s (Fig. 4B). However, in case of online hyphenation of SEC and MacroIMS, prolonged nDMA scan times precluded the detection of SEC separated species in consecutive gas‐phase electrophoresis spectra. Hence, resolution gained in SEC is lost in gas‐phase electrophoresis. In the worst case, prolonged times of gas‐phase electrophoresis can even result in information loss as SEC separated analytes are no longer detectable when analytes are electrosprayed at a time point when the corresponding EM diameter cannot pass the nDMA. Based on these results and considerations, the initial nDMA scan‐time setting of 11 s was kept for all our further experiments despite unfavorable signal shapes. It is, however, of note that for other separation problems different scan times especially between 11 and 56 s can be favorable.

### 3.3 Application of SEC–advanced nES GEMMA online hyphenation for sample desalting

As already mentioned above, nonvolatile sample components, for example, from employed buffers, are detected as analytes in gas‐phase electrophoresis. If such components are present at high concentrations, they tend to agglomerate and are detected even in the nanometer size range leading to an increase of the baseline. Furthermore, at high concentrations, salts and proteins form unspecific clusters resulting in larger EM diameters for proteins compared to analytes without salt coatings. Therefore, gas‐phase electrophoresis of samples, including residues of nonvolatile buffers, as usually employed for biological samples, is a challenging task. In most cases, offline desalting is necessary using filter membranes with corresponding pore sizes [20]. Yet, interaction of analytes with the filter material might result not just in reduction of nonvolatile buffer constituents but also in at least partial loss of analytes. The application of the introduced SEC–MacroIMS hyphenation for desalting proteinaceous analytes is therefore a high potential application.

**Figure 5:**
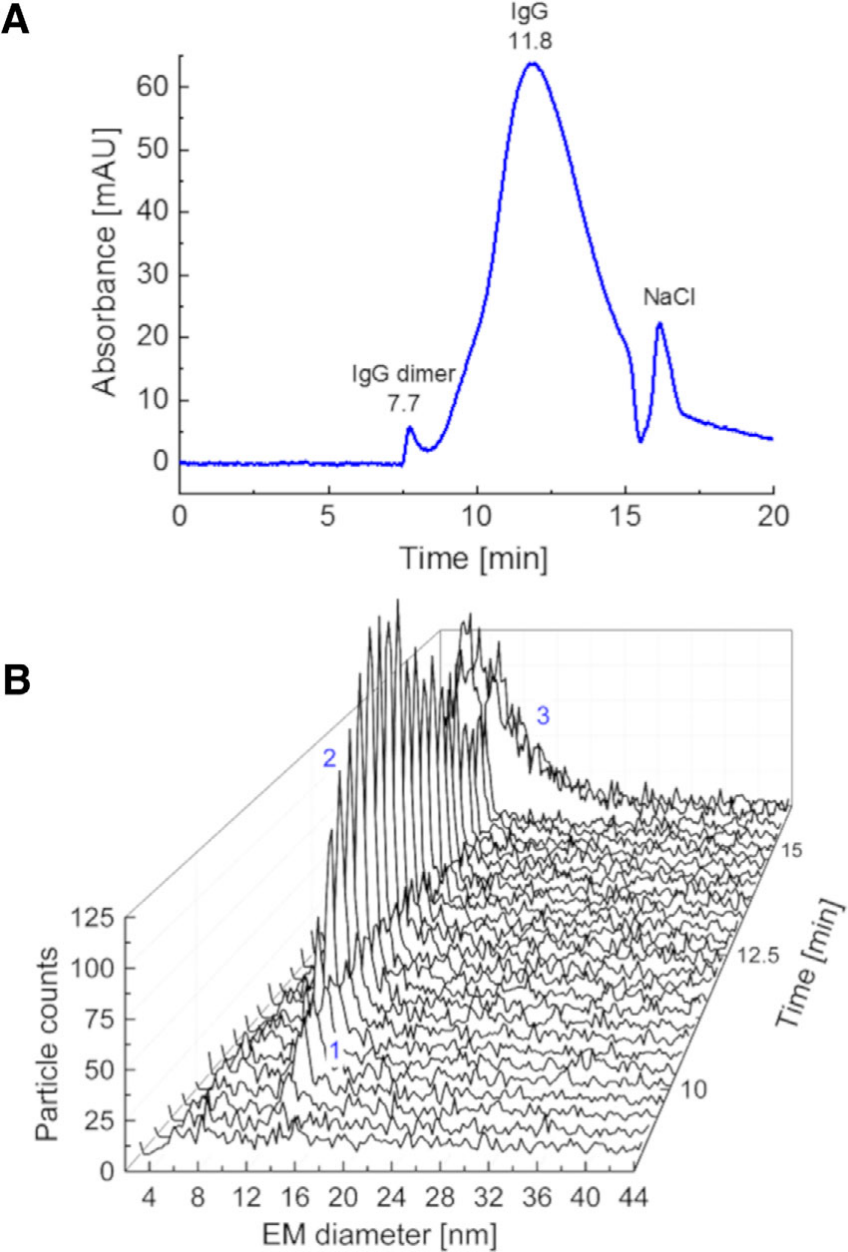
SEC/MacroIMS hyphenation enables sample desalting as exemplified by a 0.5 mg/mL IgG sample in NH_4_OAc containing 15 mM sodium chloride as nonvolatile sample component. Results for the SEC separation monitored at 210 nm (A) and the parallel MacroIMS analysis (B) are plotted. Liquid‐phase IgG dimers (1) can be separated from monomers (2) as well as sodium chloride clusters (3) in the second dimension of separation.

In Fig. 5, the separation of IgG from sodium chloride, a typical buffer constituent in physiological sample formulations, is presented. The potential of the online SEC–advanced nES GEMMA hyphenation for sample characterization is clearly demonstrated. This sample was prepared in respect to samples relevant in industry, for example, during downstream processing of biologicals. A 0.5 mg/mL IgG solution spiked with 15 mM sodium chloride was applied and the sample constituents were separated by SEC. Three signals were distinguishable already by UV detection, most likely IgG dimers and monomers and nonvolatile salt components (Fig. 5A). From 7.5 to 8.2 min, SEC exhibits only a small peak for the dimer, which is in case of IgGs a biologically relevant protein aggregation. Based on the UV signal, the ratio between liquid‐phase dimers and monomers can in principle be calculated. However, due to low particle numbers these aggregates cannot be detected via gas‐phase electrophoresis. In contrast, the elution of liquid‐phase monomers between 8.2 and 15.5 min can be confirmed via MacroIMS measurements (Fig. 5B). Gas‐phase electrophoresis relates not just to protein monomers but also dimers probably formed during the nES process. Finally, the third UV signal from SEC can be related to unspecific sodium chloride clusters yielding a heterogeneous peak in gas‐phase electrophoresis up to almost 30 nm EM diameter. Despite an increased noise level, respective baseline, in these spectra after 15.5 min elution time, the presence of sodium chloride does not interfere with the detection of IgG.

We could show that the online hyphenation of nES GEMMA with an additional separation method like SEC is a potential two‐dimensional separation system combining chromatography and electrophoresis, by this offering the possibility to measure analytes in the nanometer size range even from complex biological matrices. Loss of analytes due to adsorption of material to filters usually employed for offline buffer exchange is eliminated. Subsequently, MW values can be calculated even for native macromolecular complexes.

## 4 Concluding remarks

Size and MW determination of proteins or (bio‐)nanoparticles from biological buffers is a challenging task. For nES‐based methods nonvolatile sample components (usually to be found in concentrations far exceeding the actual (bio‐)nanoparticle concentration) significantly influence results. In the worst case, the actual particle signal is lost. Sample pretreatment to reduce the number of nonvolatile, low‐molecular mass components via any kind of filtration on the other hand, often suffers from analyte loss due to membrane interaction. We now introduce a novel online hyphenation between the LC technique SEC and gas‐phase electrophoresis on an advanced nES GEMMA device (MacroIMS). By this, liquid phase‐based aggregates could be separated from nES‐induced dimers. This now significantly improves the information relevance of gas‐phase electrophoresis measurements. For the latter, the certainty of liquid phase versus gas‐phase dimers was always under discussion and results, therefore, very often questioned.

Furthermore, this experimental setup allowed to gain insight into aggregate formation in the liquid and in the gas phase. Slight but significant changes between particles detected in these two cases indicate differences concerning the solvation state of the multimers. nDMA analysis allows to assign an EM diameter to an aggregate which was formed from surface‐dry protein monomers during the nES process while aggregates eluting from a SEC column still contain solvent molecules leading to an EM which is significantly larger (Δ EM diameter of 0.1 nm). We believe that the presented novel online SEC–MacroIMS hyphenation will break the ground for (bio‐) nanoparticle research from complex matrices and, therefore, leads to a better understanding of noncovalent protein interactions, aggregation processes, and a better understanding of biological processes where particle size plays a crucial role besides the improved basic understanding of the nES process itself.

Additionally, by this application online sample desalting is possible, providing a convenient way to overcome sample loss and aggregate formation due to filtration processes (compare, e.g., to [30] relating this problem).

*The authors have declared no conflict of interest*.

## Data Availability

The data that support the findings of this study are available from the corresponding author upon reasonable request.
